# Morphology combined with HER2 D-DISH ploidy analysis to diagnose partial hydatidiform mole: an evaluation audit using molecular genotyping

**DOI:** 10.1136/jcp-2023-209269

**Published:** 2024-03-30

**Authors:** Caroline M Joyce, Geoffrey J Maher, Susan Dineen, Nirosha Suraweera, Tommie V McCarthy, John Coulter, Keelin O'Donoghue, Michael J Seckl, Brendan Fitzgerald

**Affiliations:** 1 Pregnancy Loss Research Group, Department of Obstetrics & Gynaecology, University College Cork, Cork, Ireland; 2 Department of Biochemistry & Cell Biology, University College Cork, Cork, Ireland; 3 INFANT Research Centre, University College Cork, Cork, Ireland; 4 Trophoblastic Tumour Screening & Treatment Centre, Imperial College NHS Trust, Charing Cross Hospital, London, UK; 5 Department of Pathology, Cork University Hospital, Cork, Ireland; 6 Department of Obstetrics & Gynaecology, Cork University Maternity Hospital, Cork, Ireland

**Keywords:** genetics, immunohistochemistry, moles

## Abstract

**Aims:**

A hydatidiform mole (HM) is classified as complete (CHM) or partial (PHM) based on its morphology and genomic composition. Ancillary techniques are often required to confirm a morphologically suspected PHM diagnosis. This study sought to evaluate the clinical accuracy of PHM diagnosis using morphological assessment supported by *HER2* dual-colour dual-hapten in situ hybridisation (D-DISH) ploidy determination.

**Methods:**

Over a 2-year period, our unit examined 1265 products of conception (POCs) from which 103 atypical POCs were diagnosed as PHM or non-molar conceptuses with the assistance of *HER2* D-DISH ploidy analysis. We retrospectively audited a sample of 40 of these atypical POCs using short tandem repeat genotyping. DNA extracted from formalin-fixed paraffin-embedded tissue was genotyped using 24 polymorphic loci. Parental alleles in placental villi were identified by comparison to those in maternal decidua. To identify triploid PHM cases, we sought three alleles of equal peak height or two alleles with one allele peak twice the height of the other at each locus.

**Results:**

Thirty-six of the 40 cases (19 PHM and 17 non-molar) were successfully genotyped and demonstrated complete concordance with the original diagnosis. All PHMs were diandric triploid of dispermic origin. In two non-molar diploid cases, we identified suspected trisomies (13 and 18), which potentially explains the pregnancy loss in these cases.

**Conclusions:**

This study validates the use of *HER2* D-DISH ploidy analysis to support the diagnosis of a morphologically suspected PHM in our practice.

**WHAT IS ALREADY KNOWN ON THIS TOPIC**

**WHAT THIS STUDY ADDS**

- Ploidy determination using an adapted *HER*2 D-DISH assay assists in the diagnosis of partial hydatidiform moles in our practice.

**HOW THIS STUDY MIGHT AFFECT RESEARCH, PRACTICE OR POLICY**

- *HER2* D-DISH ploidy analysis could, if correctly implemented, improve the accuracy of HM diagnosis and reduce the reliance on centralised HM review. This approach will ensure timely access for all women to a correct diagnosis and ultimately provide more accurate HM incidence rates.

## Introduction

 Gestational trophoblastic disease (GTD) is a gynaecological condition associated with both benign and malignant entities. The most common form of GTD is a hydatidiform mole (HM) which can be a diploid diandric mole (complete hydatidiform mole, CHM) or a triploid diandric monogynic mole (partial hydatidiform mole, PHM). Malignant forms of GTD include invasive mole, choriocarcinoma, placental site trophoblastic tumour and epithelioid trophoblastic tumour, collectively known as gestational trophoblastic neoplasia (GTN). GTN may develop after any pregnancy, but the risk is greater after CHM than PHM with both HMs having a higher malignancy risk than a non-molar pregnancy. Use of ultrasound in early pregnancy has led to earlier diagnosis of HM and has been shown to correctly diagnose 88% of complete moles but only 56% of partial moles.^1^ The confirmation of HM diagnosis is made on histopathological examination of the products of conception (POCs). Early HMs can be confused with non-molar pregnancies on ultrasound and under pathological examination due to similarities in their features.^2^ It is important to establish the correct diagnosis (CHM, PHM or non-molar) as this will inform clinical management, the need for follow-up serum human chorionic gonadotrophin (hCG) monitoring and the risk of progression to GTN.^3^

HMs are characterised by excessive proliferation of syncytiotrophoblast and cytotrophoblast cells and stromal oedema.^4^ The confirmatory diagnosis of complete and partial HMs is made on the basis of morphology assessment aided by ancillary techniques, where required. A CHM is a diploid conceptus consisting solely of a paternal genome (ie, androgenetic). In CHM, the absence of a maternal genome arrests embryonic development and allows paternally expressed genes to drive trophoblastic proliferation.^5^ A diagnosis of CHM may be confirmed, if required, by the absence of immunostaining for p57^Kip2^ (p57), the product of a paternally imprinted gene (*CDKN1C*).^6^ Although useful for CHMs, p57 immunostaining is not informative when distinguishing partial mole from non-molar conceptuses. A PHM is usually a triploid conceptus consisting of one maternal genome (monogynic) and two paternal genomes (diandric) but may rarely occur as a tetraploid conceptus. The excess paternal contribution increases trophoblastic proliferation and the presence of the maternal genome allows embryonic development to proceed, often to the second trimester.^7^ It is important to classify HMs correctly as the risk of developing GTN is higher for CHM (15–20%) than for PHM (<1%) which leads to a longer hCG surveillance period for CHMs.^8^,^12^ Conversely, the risk of developing GTN after a histologically confirmed non-molar pregnancy is very low (1 in 50 000) and hCG surveillance is not required so these women can start planning a new pregnancy immediately.^13^

Diagnosis of PHM in the first trimester can be challenging when classical morphological features are subtle or absent and ancillary techniques may be required to aid diagnosis.^14^ The majority of PHMs are misdiagnosed on ultrasound as an incomplete or missed miscarriage in the first trimester.^15 16^ Diagnosis of PHM on morphology alone has reported error rates of at least 20%.^17^ Abnormal villous morphology from a hydropic non-molar placenta or conceptus with aneuploidy may mimic PHM microscopically.^18^ Karyotyping can help identify numerical and structural abnormalities, but it cannot be used on fixed tissue. In such cases, ploidy analysis using flow cytometry or in situ hybridisation (ISH) can help distinguish a diploid (aneuploid or hydropic) conceptus from a triploid conceptus (PHM).^19^,^21^ However, ploidy analysis will not distinguish between a triploid conceptus with two paternal genomes (diandric PHM) from those with two maternal genomes (digynic non-molar pregnancy).

The distribution of diandric and digynic triploidy has been reported differently depending on gestational age.^22^,^24^ Typically, digynic triploid conceptions do not display morphological features of molar pregnancy but on occasion can exhibit some focal dysmorphic features suggestive of a PHM.^7 25^ A digynic triploid conceptus may manifest on ultrasound with a growth restricted fetus, with relative macrocephaly and a non-cystic placenta but these abnormalities can be subtle and may be missed in the first trimester.^23 26^ In contrast, diandric triploid PHMs generally have an enlarged placenta with focal cystic spaces and may have an abnormal fetus.^27^ Digynic triploids have been reported to have some overlapping morphological features with PHM and as such there is a theoretical risk of overdiagnosing PHM using morphology with ploidy analysis alone; consequently, molecular genotyping has been recommended for definitive diagnosis.^28^,^30^ Evidence for the potential overlap between the morphological appearance of placental tissue from diandric and digynic triploidy is, however, sparse. Unlike PHM, digynic triploid miscarriages do not require hCG monitoring, hence the importance of obtaining an accurate diagnosis.

Molecular analysis using short tandem repeat (STR) genotyping can infer ploidy in PHMs and also identify the additional chromosomal complement.^31^ However, genotyping is not routinely available in most pathology laboratories. In this study, we sought to assess the accuracy of PHM diagnosis using morphology supported by an adapted *HER2* dual-colour dual-hapten in situ hybridisation (D-DISH) based ploidy technique by evaluation with molecular genotyping.^32^

## Materials and methods

### Study design

We performed a retrospective review of a sample of 40 atypical POCs diagnosed over a 2-year period (2018–2019) in our pathology laboratory at Cork University Hospital which is colocated with a large tertiary maternity hospital, Cork University Maternity Hospital. The audit evaluated the clinical accuracy of a combination of morphological assessment supported by *HER2* D-DISH ploidy analysis by subjecting a sample cohort of our previously diagnosed cases to STR genotyping. As we did not have access to molecular genotyping technology locally, we collaborated with colleagues in the UK and performed tissue microdissection, DNA extraction and microsatellite DNA genotyping in the Trophoblastic Tumour Screening and Treatment Centre in Imperial College London.

**Figure 1:**
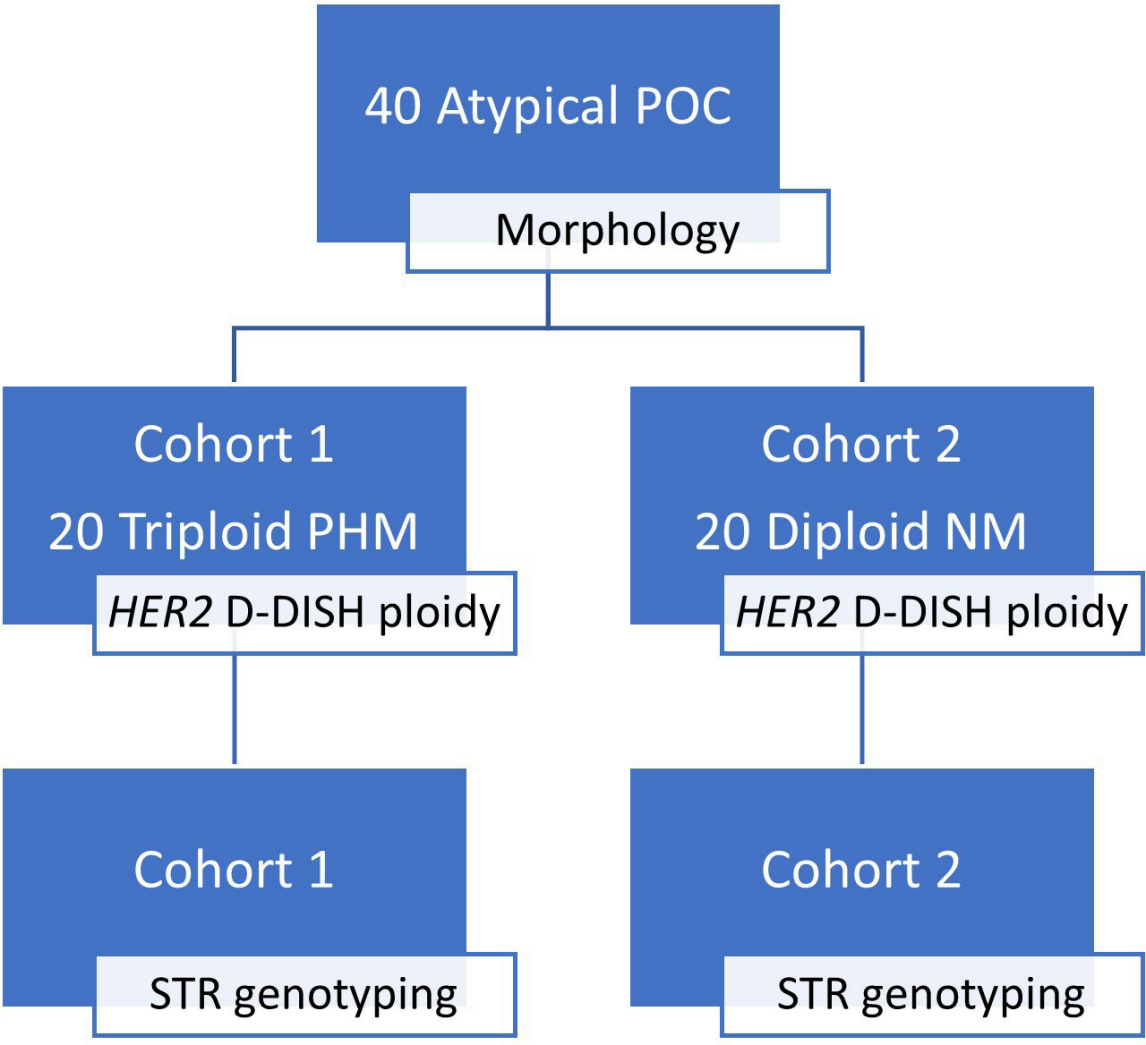
Study schematic showing case selection. D-DISH, dual-colour dual-hapten in situ hybridisation; NM, non-molar; PHM, partial hydatidiform mole; POC, product of conception; STR, short tandem repeat.

In our study period, 1265 POCs were received for pathological examination. Following initial morphological assessment, *HER2* D-DISH ploidy analysis was requested on 103 cases to assist with PHM diagnosis. Use of this technique helped diagnose almost equal numbers of triploid PHMs (50.5%, 52/103) and diploid non-molar conceptuses (49.5%, 51/103). Six PHMs were diagnosed without the assistance of ploidy determination. From these 103 atypical cases we selected 40 POCs, spread across both years, to include equal numbers of cases determined to be triploid PHM or diploid non-molar conceptuses with the support of the *HER2* D-DISH ploidy analysis technique (figure 1).^32^ Our sample cohort contained approximately one-third (38.5%, 20/52) of the triploid PHMs diagnosed with morphology and ploidy analysis during the study period, thus adequately representing samples from this timeframe. Formalin-fixed paraffin-embedded (FFPE) blocks were retrieved for each case and one H&E-stained (3 μm) section and five consecutive unstained (5 μm) sections were prepared on non-coated slides. All cases were anonymised in line with institutional ethical approval (ECM 4 (k) 9 March 2021).

### *HER2* D-DISH ploidy analysis

The VENTANA *HER2* dual ISH assay is a closed two-probe system used to determine *HER2* gene amplification status in breast and gastric carcinoma. This assay uses two-colour chromogenic ISH to enable enumeration of the ratio of *HER2* to chromosome 17 nuclear signals. We adapted this method for ploidy analysis by using the chromosome 17 enumeration probe to help classify diploid and triploid conceptuses.^32^

### Tissue microdissection and DNA extraction

**Figure 2:**
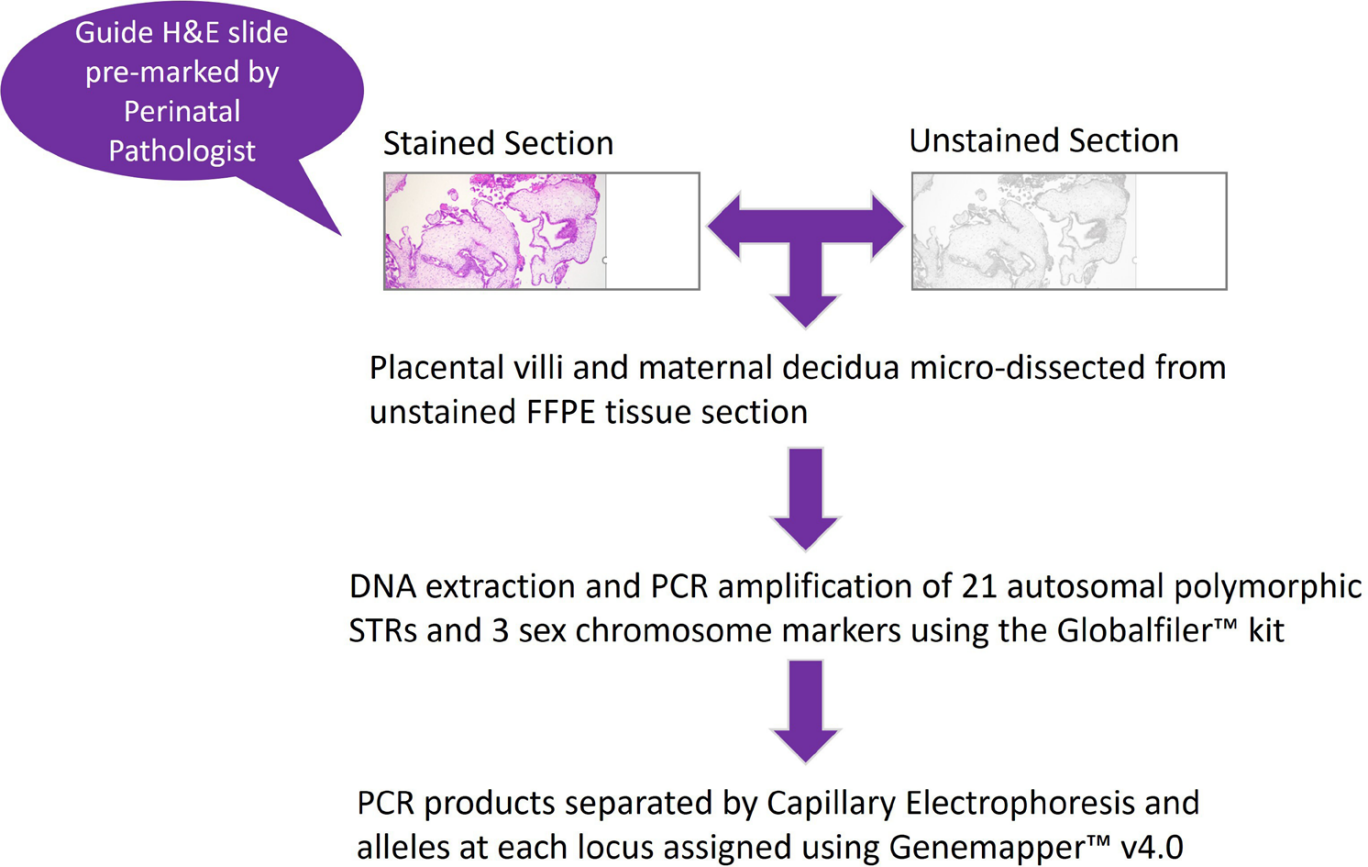
Schematic showing tissue microdissection of fixed tissue from products of conception to establish the genetic origin of hydatidiform moles by short tandem repeat (STR) genotyping. FFPE, formalin fixed paraffin embedded.

Tissue microdissection was performed using a Leica KL300 LED microscope (LeicaBiosystems.com) which filters out heat-generating infrared light and reduces the damage to heat-sensitive specimens. Single-use sterile disposable surgical scalpels, blade 21 (Swann-Morton), were used to separately dissect one area of trophoblastic villi and one area of maternal decidua from unstained slides. An adjacent H&E-stained slide premarked by a perinatal pathologist was used to guide the microdissection for each case (figure 2). The dissected tissue was placed in a prelabelled sterile Eppendorf. HistoChoice clearing agent (Sigma-Aldrich, UK) was added to remove wax from the microdissected tissue and DNA was extracted using the QIAamp DNA FFPE Tissue Kit (Qiagen, UK) according to the manufacturer’s instructions.^33^ DNA quantity was measured using the Qubit Fluorometer (Thermo Fisher) as described in the product literature.^34^

### Microsatellite DNA genotyping

Following DNA extraction from villi and decidua, polymorphic microsatellites known as STRs were amplified from DNA (1–4 ng/µL) using the GlobalFiler Multiplex PCR Amplification Kit (Applied Biosystems, UK) according to the manufacturer’s protocol.^35^ This microsatellite assay detects 21 autosomal STR loci (D3S1358, vWA, D16S539, CSF1PO, TPOX, D8S1179, D21S11, D18S51, D2S441, D19S433, TH01, FGA, D22S1045, D5S818, D13S317, D7S820, SE33, D10S1248, D1S1656, D12S391, D2S1338). It also detects three STRs on the sex chromosomes; DYS391, an insertion/deletion polymorphic marker on the Y chromosome (Y indel) and Amelogenin (a sex determination marker) on both the X and Y chromosomes.

PCR amplicons generated from villi and decidua DNA were resolved by capillary gel electrophoresis using an ABI Prism 3500 Genetic Analyser (Applied Biosystems). STR alleles were sized by comparison to a DNA ladder run adjacent to the PCR products during electrophoresis. STR alleles were assigned using GeneMapper Software V.4.0 (Applied Biosystems) according to the manufacturer’s protocol. Non-maternal (paternal) alleles were identified in trophoblastic villi by comparison to maternal alleles in decidua. Parental allele number and/or peak height ratio in the villi were used to determine ploidy for each case. Triploid conceptuses were expected to have three alleles of equal height at informative loci or two alleles with one allele peak approximately twice the height of the other (2:1 ratio).^36 37^ Diploid conceptuses were expected to have two biparental peaks of almost equal height at a locus. Preferential amplification of the shorter length allele is a feature of microsatellite genotyping and was considered when calculating allele dosage and biallelic ratios.^38^ The STR genotyping analyst was blinded to the original histopathological diagnosis.

Where low-level maternal DNA contamination was present in the villi, the genotype was inferred by adjusting peak heights to compensate for the additional maternal alleles. In such cases, the peak height of the smaller maternal allele provided a measure of the background contamination, and this was subtracted from the larger maternal peak in the contaminated sample.

### Statistical analysis

The ‘rule of 3’ power calculation was used to determine the minimum sample size required for the validation study to achieve an acceptable level of confidence in the accuracy of the results.^39^ A cohort of 40 POCs were chosen consisting of equal numbers of triploid PHM and diploid non-molar cases to yield equal power estimates of sensitivity and specificity. The sample order for POC analysis was randomised to remove any potential bias in reporting.

The accuracy of the *HER2* D-DISH ploidy assay in identifying triploid PHM conceptuses (sensitivity) and diploid non-molar conceptuses (specificity) was determined by comparison to the reference standard, molecular genotyping. A confidence interval (CI) was provided based on the number of successfully genotyped diploid and triploid conceptuses. The overall test accuracy was calculated according to the validation of qualitative tests by Mattocks *et al.*^39^

## Results

In our study cohort of women with first trimester pregnancy loss, the median maternal age was 34 years (IQR: 31–39), and the median gestational age (confirmed by sonography) was 7.3 weeks (IQR: 6.4–8.2) (table 1). The average DNA yield for the maternal decidua and trophoblastic villi was 4.0 and 3.5 ng/μL, respectively.

**Table 1:** Clinical and pathological characteristics of the study population

Clinicopathological parameters	Age	Cohort 1 (n=20)	Cohort 2 (n=20)	Total reported
Maternal age*/years	34 (31–39)			
Gestational age*/weeks	7.3 (6.4–8.2)			
Histological diagnosis: *morphology+HER2 D-DISH*		PHM (n=20)	Non-molar (n=20)	40
Genetic diagnosis: *STR genotyping*		PHM (n=19)	Non-molar (n=17)	36
Genotype		P1P2M1	PM	
Inferred ploidy		Triploid	Diploid	

* Median (interquartile rangeIQR).

D-DISH, dual-colour dual-hapten in situ hybridisation; M, maternal; M1, monogynic; P, paternal; PHM, partial hydatidiform mole; PM, Biparental; P1P2, diandric heterozygous; STR, short tandem repeat.

**Figure 3:**
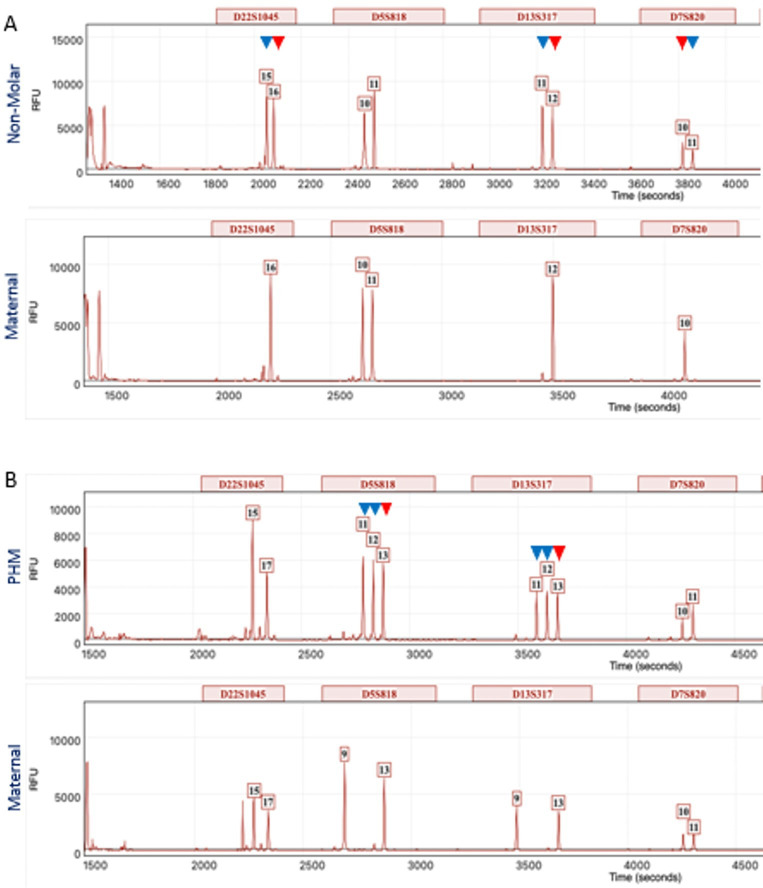
Short tandem repeat (STR) genotyping showing electropherograms for four loci in products of conception (POCs). Genotypes from placental villi are shown in the top panel with genotypes from matched maternal decidua in the lower panel. (**A**) Case 38 showing a non-molar diploid POC with biparental alleles for three loci and one non-informative locus. (**B**) Case 18 showing a triploid partial hydatidiform mole (PHM) containing three alleles at two loci (D5S818 and D13S317) and alleles in a 2:1 ratio of (inferred) paternal to maternal peak height for two loci (D22S01045 and D7S820). A blue arrowhead is used to indicate paternal alleles and a red arrowhead is used to indicate maternal alleles for informative loci. Allele nomenclature is determined by DNA fragment size on the X-axis. The Y-axis represents arbitrary units of fluorescence.

Genotypes from placental villi were compared with genotypes from matched maternal decidua for all POCs. Allele nomenclature was determined by DNA fragment size with the smallest sized fragment assigned the lowest allele number. Non-molar diploid POCs had biparental alleles at all informative loci. PHM triploid POCs had three alleles at some loci and alleles in a 2:1 ratio of (inferred) paternal to maternal peak height at other loci (figure 3).

**Figure 4:**
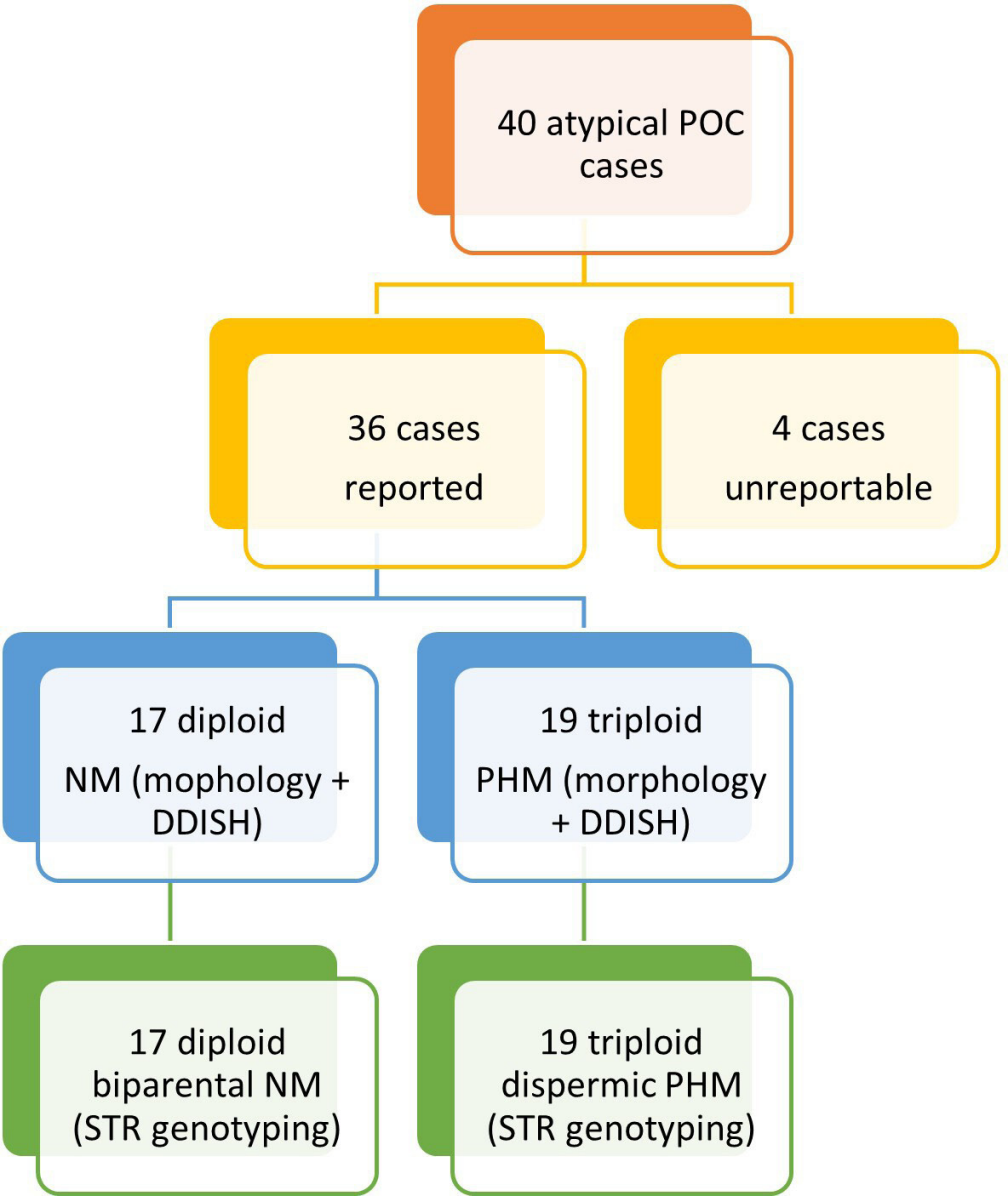
Study schematic showing results from morphology with *HER2* D-DISH and short tandem repeat (STR) genotyping. D-DISH, dual-colour dual-hapten in situ hybridisation; NM, non-molar; PHM, partial hydatidiform mole; POC, product of conception.

We successfully genotyped 36 of the 40 POCs with an STR genotype failure rate of 10% (4/40) (figure 4). STR genotypes showed complete concordance with the initial diagnosis (table 2). All 19 PHM cases were diandric triploid of dispermic origin. Two non-molar diploid cases had three alleles at a single locus which was suspicious for trisomy 13 in case 1 (D13S317) and trisomy 18 in case 5 (D18S51).

**Table 2:** STR genotyping results for 40 atypical products of conception

Case	Parental genotype and inferred ploidy	Original diagnosis	Specific loci information
2	Diandric triploid	Triploid PHM	Dispermy: D18S51, D12S91
4	Diandric triploid	Triploid PHM	Dispermy: FGA, D10S1248
8	Diandric triploid	Triploid PHM	Dispermy: D18S51, D5S818
9	Diandric triploid*	Triploid PHM	Dispermy: D18S51, D12S391
15	Diandric triploid	Triploid PHM	Dispermy: D21S11, D10S1248, D1S1656
16	Diandric triploid	Triploid PHM	Dispermy: D21S11, D22S1045
17	Diandric triploid**	Triploid PHM	Dispermy: D18S51, D22S1045
18	Diandric triploid	Triploid PHM	Dispermy: vWA, D8S1179, D5S818,D13S317, D10S1248, D1S1656
20	Possible Diandric triploid†	Triploid PHM	3 alleles at 3 loci but MD fail
22	Diandric triploid	Triploid PHM	Dispermy: vWA, D12S391
23	Diandric triploid	Triploid PHM	Dispermy: D2S441, D16S539
24	Diandric triploid	Triploid PHM	Dispermy: DS21S11, FGA
26	Diandric triploid	Triploid PHM	Dispermy: DS21S11, D1S1656
28	Diandric triploid	Triploid PHM	Dispermy: D8S1179
29	Diandric triploid	Triploid PHM	Dispermy: FGA, D13S317
34	Diandric triploid	Triploid PHM	Dispermic: D2S441
35	Diandric triploid	Triploid PHM	Dispermy: vWA, CSF1PO, D22S1045
37	Diandric triploid	Triploid PHM	Dispermy: FGA, D13S317, D1S1656
39	Diandric triploid	Triploid PHM	Dispermy: D10S1248
40	Diandric triploid	Triploid PHM	Dispermy: D18S51, FGA, D1S1656
1	Biparental diploid	Diploid NM	D13S317 (query trisomy 13)
3	Biparental diploid	Diploid NM	
5	Biparental diploid*	Diploid NM	D18S51 (query trisomy 18)
6	Biparental diploid	Diploid NM	
7	Biparental diploid	Diploid NM	
10	Biparental diploid*	Diploid NM	
11	Biparental diploid	Diploid NM	
12	Biparental diploid	Diploid NM	
13	Possible Biparental diploid*	Diploid NM	Very few informative loci
14	Biparental diploid	Diploid NM	
19	Biparental diploid	Diploid NM	
21	Biparental diploid	Diploid NM	
25	Biparental diploid*	Diploid NM	
27	Biparental diploid	Diploid NM	
30	Biparental diploid	Diploid NM	
31	Biparental diploid	Diploid NM	
32	Failed villi†	Diploid NM	
33	Failed villi†	Diploid NM	
36	Biparental diploid	Diploid NM	
38	Biparental diploid	Diploid NM	

* Genotype results post-subtraction of maternal contamination.

† Insufficient tissue/DNA.

MD, maternal decidua; NM, non-molar.

Genotype failure occurred due to scanty villi or maternal decidua in the tissue sections provided. In this study, 5% of the POCs (2/40) had limited chorionic villi (cases 32 and 33) and 2.5% (1/40) had limited maternal decidua (case 20). The case with insufficient maternal tissue had villi with a triploid genotype (three alleles at three loci) supporting a PHM classification. Additional tissue blocks were not available for these cases (following anonymisation) to allow repeat microdissection and DNA extraction. A fourth case (case 13) had very few informative markers and no marker with three alleles so DNA ploidy could not be accurately determined. The laboratory did not have access to an expanded STR genotyping assay which may have provided additional informative loci.

Our STR genotyping did not identify any false positive or false negative triploid PHM results. Accuracy and CIs were calculated using the ‘rule of 3’ estimate of statistical power for qualitative tests according to sample size.^39^ Our *HER2* D-DISH ploidy assay analysed equal numbers of diploid and triploid conceptuses, resulting in 100% sensitivity and specificity (95% CI: ≥92%, n=36).

## Discussion

Accurate classification of HMs is required for appropriate clinical management and hCG monitoring. It also dictates treatment pathways and helps predict the risk of malignant disease. Despite the availability of specific morphological criteria to guide GTD diagnosis, there is still up to 35% discordance in GTD reporting by both expert and non-expert pathologists. In a centralised pathology review, almost 95% of complete moles were confirmed but only 61% of partial moles, which highlights the need for ancillary techniques to aid diagnosis.^14 40^ Fukunaga *et al* found significant interobserver and intraobserver variability among placental pathologists when histology alone was used for diagnosis with consensus reached in only 60% (30/50) of cases.^41^ The differentiation of PHM from hydropic non-molar tissue proved the most challenging. The integration of ploidy analysis (flow cytometry) improved concordance among pathologists by 10% with consensus reached in five additional PHM cases. This review identified a need for more specific histological criteria, particularly for early HM lesions, and highlights the importance of integrated ancillary techniques to improve the accuracy of HM diagnosis.^41^,^44^ This leads to improved GTD detection rates which provides more accurate incidence rates.^45^,^47^

Ploidy analysis using our adapted *HER2* D-DISH assay will not identify the genetic origin of PHMs, therefore, there was a slight theoretical risk that a digynic triploid non-molar miscarriage could be misdiagnosed as diandric triploid PHM. In a study of 251 cases referred to the gestational trophoblastic centre in Charing Cross, the authors found that in the triploid cases, the parental contribution was paternal in 84 and maternal in only a single case, suggesting that the pathology of digynic triploidy is sufficiently different from PHM that they are recognised as non-molar by experienced pathologists.^48^ Additionally, our audit has shown that digynic triploid cases were not represented in our atypical POCs, originally diagnosed by perinatal pathologists as PHM (using morphology and ploidy analysis), and so this is not likely to be a practical concern for laboratories reporting routine specimens. Our finding of dispermy in all PHMs analysed confirmed their diandric origin and is consistent with the reported frequency of dispermic PHMs (98%) in the published literature.^49 50^

Although the risk of developing GTN after a triploid PHM is low, there have been reports of choriocarcinoma and metastatic trophoblastic disease arising after PHM.^9 11^ Some earlier studies on the risk of GTN following PHM (0.5–1%) were based on morphology with and without ploidy analysis but these studies lacked confirmatory genotyping.^10 12 43 51 52^ Recent publications using STR genotyping to confirm PHM suggest the risk of progression to malignancy may be much lower.^9 11 53^ Scholz *et al* provide an extremely low risk (0%) of progression from PHM to GTN based on four cohort studies with 265 patients.^54^ Clinical guidelines for GTD management therefore recommend a shorter hCG surveillance period after PHM.^55^ Given the lack of consensus on the exact risk of GTN after PHM, patient preference should be considered when choosing chemotherapy, as some PHM cases with persistent disease have normalised without treatment.^56^

In our study of first trimester pregnancy loss POCs, the median gestational age was 7.3 weeks. At this early stage in gestation, some of the classical morphological features of PHM may be subtle or absent on pathology and use of adjunct tools to determine ploidy can greatly assist the diagnosis of PHM. Due to the widespread use of ISH in pathology laboratories, there should be easy access to ancillary techniques such as *HER2* D-DISH for atypical/early PHMs to confirm triploidy and exclude PHM mimics.

Given that *HER2* D-DISH nuclear count signals are quantified from chromosome 17 centromeric probes, there is a possibility that trisomy 17, although rarely encountered in pregnancy loss,^57^,^59^ could be misinterpreted as triploidy if *HER2* D-DISH was used in isolation. Our study was limited by the fact that the STR amplification kit used did not include STRs on chromosome 17. While we were unable to exclude the presence of trisomy 17 in our study cohort, PHM was confirmed as triploid based on genotyping of other chromosomes, excluding the possibility of isolated trisomy 17. Trisomy 17 has never been observed in live births and is only found in 1 in 1000 miscarriages.^60^ Also, there is a lack of evidence that trisomy 17 may mimic PHM during initial morphological assessment. Given both of these latter factors, the theoretical risk of misinterpreting trisomy 17 as a PHM during routine practice would seem to be extremely low.

Another limitation of the study is that ISH may be affected by weak staining, potentially leading to undercounting of nuclear signals, and resulting in a missed triploid conceptus. In our practice, cases with equivocal scores due to poor staining are restained to achieve better staining and are often then scored successfully.^32^ Some centres use genotyping when weak or equivocal staining is encountered and this practice could be adopted in laboratories with access to STR genotyping. In our audit, all cases initially determined to be diploid based on *HER2* D-DISH were confirmed diploid by molecular genotyping yielding no false negatives in our study cohort.

Adoption of molecular genotyping in suspected cases of PHM to infer ploidy may also detect clinically relevant aneuploidy in non-molar pregnancies and thus provide an additional explanation for the pregnancy loss.^18 61^ In our study, two non-molar diploid cases were identified with suspected trisomy (13 and 18). Trisomy 13 has some common features with HM on ultrasound and three cases mimicking PHM have been reported.^62^,^64^

While STR genotyping is an extremely valuable technique, there are some challenges to its application in pathology. Occasionally, scant villous tissue in the first trimester may preclude the use of genotyping to aid PHM diagnosis. Another challenge is the possibility of maternal DNA contamination in trophoblastic villi and vice versa. Dissection of villi for genotyping is technically difficult and contamination of villi with maternal decidua can complicate the interpretation of genotyping results. Clinical practice guidelines recommend dissecting two or more villous regions for each POC to exclude mosaicism (ie, two diploid cell lines) and improve the accuracy of genotype interpretation.^14 38^ Another limiting factor is the quality of DNA from FFPE tissue which is often degraded due to chemical fixation and this can produce a low DNA yield as occurred in two of our cases. A further limitation is the lack of sufficient informative loci in a conceptus to establish parental origin. In practice, at least one fully informative locus (three different parental alleles) with other loci supporting a finding of triploidy (2:1 ratio of paternal to maternal peak heights) is desirable.^38^

While the financial costs of reagent purchase for ISH and molecular genotyping equipment are comparable, the need for additional scientific expertise to interpret genotyping data may restrict the adoption of genotyping in smaller pathology laboratories. In contrast, many laboratories have access to ISH. Therefore, implementation of the adapted *HER2* D-DISH assay and bespoke ‘rule of 5’ scoring system for ploidy analysis should not necessitate additional investment.^32^ The integration of ploidy analysis into PHM diagnosis will help improve the accuracy of diagnosis and reduce the reliance on centralised HM review. This approach will provide equity of access for all women to reliable diagnostic services and eliminate delays in the diagnosis of GTD, a highly curable gynaecological disorder.

## Conclusion

It is sometimes stated that morphology with ploidy analysis alone will not definitively differentiate PHM. This is because ploidy determination does not identify the parental origin of the additional genome leading to a theoretical risk of overdiagnosing digynic triploidy as PHM. In our practice, morphological assessment by an experienced perinatal pathologist supported by *HER2* D-DISH ploidy analysis does not yield false positive results due to digynic triploidy. Instead, it proves to be a reliable adjunct to HM morphological diagnosis, potentially contributing to more accurate incidence rates. Larger prospective studies from other units would be useful to determine whether digynic triploidy causes morphological confusion with PHM in others’ practice or whether this risk is mostly theoretical. Our study emphasises the importance of the initial morphological interpretation by the pathologist. Although morphological interpretation in HM diagnosis can be challenging, it is still the starting point for nearly all routine tissue diagnostics. In our practice, such morphological assessment appears to screen out cases of digynic triploidy. Appropriate use of ploidy determination as an adjunct tool then provides an efficient method of assistance in confirming or excluding PHM. Such an approach should be achievable in many laboratories where it should serve to improve diagnostic confidence, particularly where access to molecular genotyping is limited.

## Data Availability

All data relevant to the study are included in the article or uploaded as supplementary information.
